# Childhood diarrhoeal deaths in seven low- and middle-income countries

**DOI:** 10.2471/BLT.13.134809

**Published:** 2014-06-23

**Authors:** Ahmed Ehsanur Rahman, Md Moinuddin, Mitike Molla, Alemayehu Worku, Lisa Hurt, Betty Kirkwood, Sanjana Brahmawar Mohan, Sarmila Mazumder, Zulfiqar Bhutta, Farrukh Raza, Sigilbert Mrema, Honorati Masanja, Daniel Kadobera, Peter Waiswa, Rajiv Bahl, Mike Zangenberg, Lulu Muhe, on behalf of the Persistent Diarrhoea Research Group

**Affiliations:** a International Centre for Diarrhoeal Disease Research, Dhaka, Bangladesh.; b College of Health Sciences, Addis Ababa University, Addis Ababa, Ethiopia.; c London School of Hygiene and Tropical Medicine, London, England.; d Centre for Health Research and Development of the Society for Applied Studies, New Delhi, India.; e Aga Khan University, Karachi, Pakistan.; f Ifakara Health Institute, Ifakara and Rufiji, United Republic of Tanzania.; g Makerere University, Iganga/Mayuge Health and Demographic Surveillance Site, Iganga, Uganda.; h Department of Maternal, Newborn, Child and Adolescent Health, World Health Organization, Avenue Appia 20, 1211 Geneva 27, Switzerland.

## Abstract

**Objective:**

To investigate the clinical characteristics of children who died from diarrhoea in low- and middle-income countries, such as the duration of diarrhoea, comorbid conditions, care-seeking behaviour and oral rehydration therapy use.

**Methods:**

The study included verbal autopsy data on children who died from diarrhoea between 2000 and 2012 at seven sites in Bangladesh, Ethiopia, Ghana, India, Pakistan, Uganda and the United Republic of Tanzania, respectively. Data came from demographic surveillance sites, randomized trials and an extended Demographic and Health Survey. The type of diarrhoea was classified as acute watery, acute bloody or persistent and risk factors were identified. Deaths in children aged 1 to 11 months and 1 to 4 years were analysed separately.

**Findings:**

The proportion of childhood deaths due to diarrhoea varied considerably across the seven sites from less than 3% to 30%. Among children aged 1–4 years, acute watery diarrhoea accounted for 31–69% of diarrhoeal deaths, acute bloody diarrhoea for 12–28%, and persistent diarrhoea for 12–56%. Among infants aged 1–11 months, persistent diarrhoea accounted for over 30% of diarrhoeal deaths in Ethiopia, India, Pakistan, Uganda and the United Republic of Tanzania. At most sites, more than 40% of children who died from persistent diarrhoea were malnourished.

**Conclusion:**

Persistent diarrhoea remains an important cause of diarrhoeal death in young children in low- and middle-income countries. Research is needed on the public health burden of persistent diarrhoea and current treatment practices to understand why children are still dying from the condition.

## Résumé

**Objectif:**

Étudier les caractéristiques cliniques des enfants qui sont morts de maladies diarrhéiques dans des pays à revenu faible et intermédiaire, telles que la durée de la diarrhée, les conditions de comorbidité, le comportement en matière de sollicitation des soins et l'utilisation de thérapie de réhydratation orale.

**Méthodes:**

L'étude a inclus les données des autopsies verbales sur des enfants décédés de maladies diarrhéiques entre 2000 et 2012 dans 7 sites du Bangladesh, de l'Éthiopie, du Ghana, de l'Inde, du Pakistan, de l'Ouganda et de la Tanzanie, respectivement. Les données provenaient des sites de surveillance démographique, des essais randomisés et d'une enquête démographique et sanitaire étendue. Les diarrhées ont été classées par type, en tant que diarrhée aqueuse aiguë, diarrhée sanglante aiguë ou diarrhée persistante, et les facteurs de risque ont été identifiés. Les décès chez les enfants âgés de 1 à 11 mois et de 1 à 4 ans ont été analysés séparément.

**Résultats:**

Le pourcentage de la mortalité infantile liée aux maladies diarrhéiques varie considérablement entre les 7 sites, de moins de 3% à 30%. Chez les enfants âgés de 1 à 4 ans, les diarrhées aqueuses aiguës représentaient 31% à 69% des décès liés aux maladies diarrhéiques, les diarrhées sanglantes aiguës 12% à 28% et les diarrhées persistantes 12% à 56%. Chez les enfants âgés de 1  mois, les diarrhées persistantes représentaient plus de 30% des décès liés aux maladies diarrhéiques en Éthiopie, en Inde, en Ouganda et en Tanzanie. Dans la plupart des sites, plus de 40% des enfants qui sont morts de diarrhée persistante souffraient de malnutrition.

**Conclusion:**

La diarrhée persistante reste une cause importante de décès liés aux maladies diarrhéiques chez les jeunes enfants dans les pays à revenu faible et intermédiaire. Il est nécessaire de mener des recherches sur la charge de la diarrhée persistante pour la santé publique et sur les pratiques actuelles de traitement afin de comprendre pourquoi des enfants meurent encore de cette maladie.

## Resumen

**Objetivo:**

Investigar las características clínicas de los niños que murieron de diarrea en países de ingresos medios y bajos, tales como la duración de la diarrea, las afecciones comórbidas, el comportamiento de búsqueda de atención y el uso de la terapia de rehidratación oral.

**Métodos:**

El estudio incluyó datos verbales de autopsias de niños que murieron de diarrea entre los años 2000 y 2012 en siete emplazamientos en Bangladesh, Etiopía, Ghana, India, Pakistán, Uganda y la República Unida de Tanzanía, respectivamente. Los datos provinieron de centros de vigilancia demográfica, ensayos aleatorios y una encuesta demográfica y de salud ampliada. El tipo de diarrea se clasificó como acuosa aguda, sanguinolenta aguda o persistente, y se identificaron los factores de riesgo. Se analizaron por separado las muertes en niños de 1 a 11 meses y de 1 a 4 años.

**Resultados:**

La proporción de muertes infantiles por diarrea varió considerablemente entre los siete emplazamientos, de menos del 3 % hasta el 30 %. Entre los niños de entre 1 y 4 años, la diarrea acuosa aguda representó del 31 % al 69 % de las muertes por diarrea, la diarrea sanguinolenta aguda, del 12 % al 28 %, y la diarrea persistente, del 12 % al 56 %. Entre los niños de 1 a 11 meses, la diarrea persistente supuso más del 30 % de las muertes por diarrea en Etiopía, India, Pakistán, Uganda y la República Unida de Tanzanía. En la mayoría de los emplazamientos, más del 40 % de los niños que murieron por diarrea persistente estaban malnutridos.

**Conclusión:**

La diarrea persistente sigue siendo una causa importante de muerte diarreica en niños pequeños en países de ingresos medios y bajos. Es necesario realizar investigaciones sobre la carga de salud pública de la diarrea persistente y las prácticas de tratamiento actuales para entender por qué los niños siguen muriendo por esta afección.

## ملخص

**الغرض:**

تحري الخصائص السريرية للأطفال الذين لقوا حتفهم جراء الإصابة بالإسهال في البلدان المنخفضة والمتوسطة الدخل، مثل مدة الإسهال واعتلالات المراضة المشتركة وسلوك الحصول على الرعاية واستعمال المعالجة بالإمهاء الفموي.

**الطريقة:**

شملت الدراسة بيانات التشريح الشفوي عن الأطفال الذين لقوا حتفهم جراء الإصابة بالإسهال بين عامي 2000 و2012 في سبعة مواقع في بنغلاديش وإثيوبيا وغانا والهند وباكستان وأوغندا وجمهورية تنزانيا المتحدة، على التوالي. وتم الحصول على البيانات من مواقع الترصد الديمغرافي والتجارب العشوائية ومن مسح ديمغرافي وصحي موسع. وتم تصنيف نوع الإسهال إلى إسهال مائي حاد أو إسهال دموي حاد أو إسهال مستمر وتم تحديد عوامل الاختطار. وتم تحليل الوفيات لدى الأطفال الذين تتراوح أعمارهم من شهر إلى 11 شهراً ومن سنة إلى 4 سنوات بشكل منفصل.

**النتائج:**

اختلفت نسبة وفيات الأطفال جراء الإصابة بالإسهال بشكل كبير بين المواقع السبعة من أقل من 3 % إلى 30 %. ومن بين الأطفال في سن 1-4 سنوات، تعزى نسبة 31 % إلى 69 % من الوفيات جراء الإسهال إلى الإسهال المائي الحاد ومن 12 % إلى 28 % إلى الإسهال الدموي الحاد ومن 12 % إلى 56 % إلى الإسهال المستمر. تجاوزت الوفيات جراء الإصابة بالإسهال 30 % في إثيوبيا والهند وباكستان وأوغندا وجمهورية تنزانيا المتحدة بين الرضع في سن 1-11 شهراً. وكان أكثر من 40 % من الأطفال الذين لقوا حتفهم جراء الإصابة بالإسهال المستمر يعانون من سوء التغذية في معظم المواقع.

**الاستنتاج:**

يظل الإسهال المستمر سبباً مهماً للوفاة جراء الإسهال لدى صغار الأطفال في البلدان المنخفضة والبلدان المتوسطة الدخل. ويجب إجراء بحوث حول عبء الإسهال المستمر في الصحة العمومية وممارسات العلاج الحالية بغية فهم سبب استمرار وفاة الأطفال جراء الإصابة بهذا الاعتلال.

## 摘要

**目的:**

调查中低收入国家死于腹泻的儿童临床特征，如腹泻时长、共患病情况、求医行为和口服补液疗法使用。

**方法:**

研究包括在2000年到2012年之间在孟加拉国、埃塞俄比亚、加纳、印度、巴基斯坦、乌干达和坦桑尼亚联合共和国七个地点死于腹泻病的儿童的口头尸检数据。数据来自人口监测点、随机试验和扩展的人口统计和健康调查。腹泻的类型分为急性水样腹泻、急性出血或持续腹泻，并进行风险因素识别。对1至11个月和1到4岁的儿童死亡分别进行分析。

**结果:**

七个地点死于腹泻儿童的比例差别很大，从低于3%到30%不等。在1到4岁的儿童腹泻死亡的原因中，急性水样腹泻占31%到69%、急性出血性腹泻占12%至28%，持续腹泻占12%到56%。在埃塞俄比亚、印度、巴基斯坦、乌干达和坦桑尼亚联合共和国，1到11个月婴儿中持续腹泻是30%以上腹泻死亡的原因。在大多数地点，超过40%死于持续腹泻的儿童营养不良。

**结论:**

持续性腹泻仍然是中低收入国家幼儿腹泻死亡的一个重要原因。需要对持续腹泻的公共卫生负担和目前的治疗实践进行研究，从而理解儿童仍死于这种疾病的原因。

## Резюме

**Цель:**

Исследовать клинические характеристики детей, умерших от диареи, в странах с низким и средним уровнем дохода, такие как продолжительность диареи, сопутствующие заболевания, активное обращение за медицинской помощью и использование пероральной регидратационной терапии.

**Методы:**

В ходе исследования были рассмотрены данные, полученные путем опроса очевидцев об обстоятельствах смерти детей от диареи за 2000-2012 гг. в семи странах: Бангладеше, Эфиопии, Гане, Индии, Пакистане, Уганде и Объединенной Республики Танзании соответственно. Данные поступали из мест проведения демографических наблюдений, рандомизированных исследований и расширенного Опроса, посвященного демографии и здравоохранению. По типу диарея была классифицирована как острая водянистая, острая геморрагическая или упорная. Также были выявлены факторы риска. Был проведен отдельный анализ смертности среди детей в возрасте от 1 до 11 месяцев и от 1 года до 4 лет.

**Результаты:**

Соотношение детских смертей из-за диареи значительно отличалось в семи центрах и варьировались от менее 3% до 30%. Среди детей в возрасте от 1-го года до 4-х лет 31-69% смертей от диареи приходились на острую водянистую диарею, от 12% до 28% — на острую геморрагическую диарею и от 12% до 56% — на упорную диарею. Среди детей в возрасте от 1 до 11 месяцев на упорную диарею приходилось более 30% смертей от диареи в Эфиопии, Индии, Пакистане, Уганде и Объединенной Республике Танзании. В большинстве стран более 40% детей, умерших от упорной диареи, страдали от недоедания.

**Вывод:**

Упорная диарея остается важной причиной смерти от диареи у детей младшего возраста в странах с низким и средним уровнем дохода. Чтобы понять, почему дети продолжают умирать от этого заболевания, необходимы исследования бремени упорной диареи для общественного здравоохранения и существующей практики лечения.

## Introduction

In the 1980s, five million children worldwide died every year because of diarrhoea, essentially because there was no readily available treatment.^1^ In the intervening 30 years, improved management of diarrhoea, such as treatment with oral rehydration solutions, intravenous fluids and zinc,^2^ has led to a substantial reduction in mortality to approximately 614 000 deaths every year.^3^^,^^4^ Nevertheless, diarrhoea remains a common cause of death in all children and is the second most common cause in those aged over 1 month.^5^^,^^6^ It is worth asking why children continue to die from the condition.

Diarrhoeal diseases can be classified according to their clinical pattern as: (i) persistent diarrhoea (i.e. diarrhoea lasting 14 days or more); (ii) acute watery diarrhoea (i.e. diarrhoea without blood lasting less than 14 days); or (iii) acute bloody diarrhoea (i.e. diarrhoea with blood lasting less than 14 days).^7^ With acute diarrhoea, dehydration is the main contributor to mortality and treatment with oral rehydration solutions and zinc is effective. However, persistent diarrhoea is associated with malnutrition, delayed growth and development, vitamin A deficiency and systemic infections such as respiratory infections and urinary tract infection,^8^^,^^9^ which makes treatment more complex.

Following the recent publication of the Global Action Plan for the Prevention and Control of Pneumonia and Diarrhoea,^10^ there was a renewed emphasis on the management of diarrhoea. In addition, diarrhoea is also a key feature of initiatives such as the United Nations Commission on Life-Saving Commodities for Women's and Children's Health and the Commission on Information and Accountability for Women's and Children's Health. These global strategies focus mainly on the treatment of acute diarrhoea using oral rehydration and zinc. Although these medications are key components in the treatment of diarrhoea, there is no mention of specific treatment for persistent diarrhoea. The aim of the current study was to identify: (i) conditions underlying childhood diarrhoea; (ii) gaps in the management of childhood diarrhoea; and (iii) associations between death due to childhood diarrhoea and clinical characteristics such as the type of diarrhoea, comorbid conditions, care-seeking behaviour and the use of oral rehydration therapy.

## Methods

The study involved verbal autopsy data on children from low- and middle-income countries who died because of diarrhoea. All sites in the INDEPTH network were invited to participate and were asked if they were able to provide data on at least 50 diarrhoeal deaths in children less than 5 years of age during the period 2000 to 2012.^11^ Only population-based studies were included and both demographic surveillance and randomized cohort studies were eligible. Seven sites were able to provide sufficient verbal autopsy data: they were in Bangladesh, Ethiopia, Ghana, India, Pakistan, Uganda and the United Republic of Tanzania (Table 1).

**Table 1:** Review of childhood diarrhoeal deaths, population-based studies in seven countries, 2000–2012

Study characteristic	Bangladesh	Ethiopia	Ghana	India	Pakistan	Uganda	United Republic of Tanzania
Population covered	225 202	62 178	600 000	601 320	160 493 000	69 243	222 958
Time period	2003–2011	2003–2012	2003–2008	2008–2010	2006–2007	2007–2010	2000–2011
Type of surveillance	Demographic surveillance	Demographic surveillance	Randomized, placebo-controlled trial	Randomized trial	Demographic and Health Survey	Demographic surveillance	Demographic surveillance
Frequency of surveillance	Bimonthly	Quarterly	4 weekly	Quarterly	Every 5 to 10 years	Biannually	Quarterly
Age range of children monitored for this analysis, months	1–59	1–59	1–11	1–11	1–59	1–59	1–59

DHS: demographic and health survey.

The study included data on all children aged 1 to 59 months in every household covered by the sites in Bangladesh, Ethiopia, Pakistan, Uganda and the United Republic of Tanzania. For Ghana, data were available on all infants aged 1 to 11 months who were enrolled in a vitamin A trial.^12^ For India, only infants aged 1 to 11 months from the control arm of the Integrated Management of Neonatal and Childhood Illness trial were included because the trial intervention could have affected the risk of death due to diarrhoea.^13^ Neonates were not included because their clinical presentation was different and limited data were available. All sites used a common framework and a standardized questionnaire for conducting verbal autopsies;^14^ the cause of death was assigned by two independent physicians and, if there was a disagreement, a third physician carried out a review. A data collection template was developed to standardize the variables extracted from verbal autopsy data across the different sites. The variables selected for each child were: age; gender; duration and type of diarrhoea; the presence of blood in stool; malnutrition (i.e. the child was either wasted or very thin or had swollen ankles or yellowish hair); treatment with oral rehydration solutions or intravenous fluids; and the caregiver seeking health care at a facility. Also variables on the prevalence of human immunodeficiency virus (HIV) infection, treatment with zinc or other medicines and the duration of diarrhoea before treatment was started were selected.

In Bangladesh, data came from the rural subdistrict of Matlab where information on child deaths was registered by a health and demographic surveillance system established by the International Centre for Diarrhoeal Disease Research in Bangladesh between 2003 and 2011.^15^ In 2003 and 2004, only one of the two reviewers was a trained physician but from 2005 onwards the cause of death was assigned by a physician. In Ethiopia, data were collected between 2003 and 2012, at a demographic surveillance site established by the Butajira Rural Health Programme in nine rural and one urban *kebele* (i.e. administrative unit) in the former Meskan and Mareko district.^16^ Data on vital status and migration were registered quarterly and verbal autopsies have been conducted since 2003. In Ghana, part of the cohort data came from a cluster-randomized, double-blind, placebo-controlled vitamin A trial that ran between 2000 and 2008. Verbal autopsy data were available for child deaths between 2003 and 2008. The trial was performed in seven predominantly rural districts to assess the effect of weekly, low-dose, vitamin A supplementation in women and found that supplementation did not affect all-cause or diarrhoeal mortality in women or their children. Households were visited every 4 weeks and the single most important cause of death was assigned by physicians.^12^

In India, the study site was taking part in a cluster-randomized cohort study in which the package of interventions that formed part of the Integrated Management of Neonatal and Childhood Illness strategy was compared with no intervention. The study was conducted between 2008 and 2010 and included infants less than 1 year of age. All households were visited by field workers every month to identify new pregnancies and to inquire about the outcome of previously identified pregnancies. Households where a live birth had taken place were visited 29 days after the birth and, subsequently, every quarter to document the vital status of the infant and to conduct a verbal autopsy if appropriate.^13^ In Pakistan, data were collected, and verbal autopsies on child deaths were carried out, in an extended Demographic and Health Survey.^17^ For this analysis, data came from the Pakistan Demographic and Health Survey for 2006 to 2007. In the United Republic of Tanzania, the Ifakara Health Institute has been implementing a health and demographic surveillance system at two sentinel sites.^18^ Households are visited three times a year to carry out health surveillance and verbal autopsies and data were obtained between 2000 and 2011. In Uganda, the Iganga–Mayuge Health and Demographic Surveillance Site comprised 65 villages drawn from Iganga and Mayuge districts and every household was visited twice a year.^19^ Village scouts reported all births and deaths in their villages and trained interviewers conducted verbal autopsies. Data were obtained between 2007 and 2010.

Our analysis included only deaths for which the underlying or single cause of death was diarrhoea. Information from verbal autopsies was used to identify risk factors for death and to classify the type of diarrhoea as either: (i) persistent; (ii) acute watery; or (iii) acute bloody diarrhoea. In addition, deaths in two age groups were studied: postneonatal infants aged 1 to 11 months and children aged 1 to 4 years at death. For each study site, the proportion of diarrhoeal deaths due to each type of diarrhoea was calculated and the presence of the following risk factors was determined from information provided by caregivers: malnourishment; receipt of oral rehydration solutions or intravenous fluids during the final illness; seeking health care outside the home during the final illness; and the sex of the child for whom care was sought. Insufficient information was available to determine whether the type of diarrhoea varied according to either the prevalence of HIV infection, treatment with zinc or other medicines or the duration of diarrhoea before treatment was started.

## Results

The study included data on childhood deaths collected between 2000 and 2012. The population covered at the seven study sites ranged from approximately 60 000 to 600 000, except for Pakistan where the Demographic and Health Survey covered the country’s total population of 160 493 000 (Table 1). The overall infant mortality rate reported by the World Health Organization (WHO) and our estimates of mortality rates in our two age groups were comparable across the seven sites (Table 2);^20^ rates were highest in Ethiopia and Pakistan. However, there was a considerable difference in the rate of use of oral rehydration reported in demographic and health surveys: from 26.0% in India to 77.6% in Bangladesh.^12^^,^^13^^,^^15^^–^^19^^,^^21^

**Table 2:** Review of childhood diarrhoeal deaths, population-based studies in seven countries, 2008^a^

Characteristics	Bangladesh	Ethiopia	Ghana	India	Pakistan	Uganda	United Republic of Tanzania
Infants less than12 months of age, no.	4 779	1 465	14 485	NA	NA	1 948	3 196
Children less than 5 years of age, no.	24 226	7 583	NA	NA	1 019 533^b^	11 073	28 708
Live births, no.	5 038	1 415	15 372^c^	13 897	3 830 973^b^	2 185	7 118
Deaths among infants aged 1 to 11 months, no.	179	76	320^c^	226	288 192^b^	39	204
Deaths among children aged 1 to 59 months, no.	241	139	NA	NA	365 729^b^	120	270
Mortality rate in infants aged 1 to 11 months, per 1 000 live births	35.5	53.7	20.8	16.3	75.2	17.8	28.7
Mortality rate in children aged 1 to 59 months, per 1 000	47.8	98.2	NA	NA	95.5	54.9	37.9
WHO infant mortality rate, per 1 000 live births^d^	43	69	51	52	72	84	67
Proportion of children with diarrhoea who received oral rehydration solutions, %^e^	77.6^f^	26.3^g^	44.5^h^	26.0^i^	41.1^j^	43.5^g^	44.0^f^

NA: not available; WHO: World Health Organization.

^a^ Data are for 2008 except where otherwise stated.

^b^ Data for Pakistan came from the 2006–2007 Demographic and Health Survey.

^c^ The value is for 2007.

^d^ Data are from *World health statistics 2010*.^20^

^e^ Data are from demographic and health surveys.

^f^ Data for 2010.

^g^ Data for 2011.

^h^ Data for 2007 or 2008.

^i^ Data for 2005–2006.

^j^ Data for 2006–2007.

In Bangladesh, the study site covered a population of 225 202 and verbal autopsies were carried out between 2003 and 2011 on 2138 deaths in children aged 1 to 59 months (Table 3). Diarrhoea was a direct cause of death in 59 cases: 41 in children aged 1 to 11 months and 18 in those aged 1 to 4 years. In Ethiopia, the population covered was 62 178 and 681 children aged 1 to 59 months died between 2003 and 2012, including 60 who died because of diarrhoea: 28 aged 1 to 11 months and 32 aged 1 to 4 years. In Ghana, the study population was 600 000 and 153 of 1790 deaths in children aged 1 to 11 months enrolled in the trial were due to diarrhoea. Complete data, which enabled the type of diarrhoea to be classified, were available for only 145 of these deaths. In India, 197 deaths due to diarrhoea were recorded among 809 deaths in infants aged 1 to 11 months in the control arm of the randomized study. In Pakistan, data were available from the Demographic and Health Survey for 2006 to 2007 on 1426 deaths among children aged 1 to 59 months, of which 318 were due to diarrhoea: 220 in infants aged 1 to 11 months and 98 in children aged 1 to 4 years. In Uganda, the surveillance site covered a population of 69 243 and 631 deaths were recorded in children less than 5 years of age between 2007 and 2010: diarrhoea was the cause of death in 115 cases. Due to a lack of information on disease duration, the analysis included only 77 of the 115: 28 out of 46 infants (1–11 months) and 49 out of 115 children (1– 4 years). In the United Republic of Tanzania, the site covered a population of 222 958 and 3774 deaths were registered among children less than 5 years of age between 2000 and 2011, of which 80 were due to diarrhoea: 39 in infants aged 1 to 11 months and 41 in children aged 1 to 4 years.

**Table 3:** Review of childhood diarrhoeal deaths, population-based studies in seven countries, 2000–2012

Characteristic	Bangladesh	Ethiopia	Ghana	India	Pakistan^a^	Uganda	United Republic of Tanzania
Study period	2003–2011	2003–2012	2003–2008	2008–2010	2006–2007	2007–2010	2000–2011
Deaths among infants aged 1 to 11 months, no.	1665	95	1790	809	862	305	1715
Deaths among children aged 1 to 59 months, no.	2138	681	NA	NA	1426	631	3774
Diarrhoeal deaths among infants aged 1 to 11 months, no. (% of all deaths)	41 (2.5)	28 (29.5)	153 (8.5)	197 (24.4)	220 (25.5)	46 (15.1)	39 (2.3)
Diarrhoeal deaths among children aged 1 to 59 months, no. (% of all deaths)	59 (2.8)	60 (8.8)	NA	NA	318 (22.3)	115 (18.2)	80 (2.1)

NA: not available.

^a^ Data on diarrhoeal deaths in Pakistan were available only for a subset of the 2006–2007 Demographic and Health Survey.

The proportion of all deaths in infants aged 1 to 11 months that were due to diarrhoea varied considerably across the seven countries: from less than 3% in Bangladesh and the United Republic of Tanzania to between 24% and 30% in Ethiopia, India and Pakistan (Table 3). At most sites, the proportion of deaths due to diarrhoea was similar in infants aged 1 to 11 months and in children aged 1 to 4 years. The exception was Ethiopia where diarrhoea accounted for 29.5% (28/95) of deaths among infants aged 1 to 11 months but only 8.8% (60/681) among children aged 1 to 4 years.

The type of diarrhoea involved also varied greatly between countries (Table 4). Among infants aged 1 to 11 months, persistent diarrhoea accounted for 30% or more of diarrhoeal deaths in Ethiopia, India, Pakistan, Uganda and the United Republic of Tanzania and for more than 35% in three of these countries. Among children aged 1 to 4 years, persistent diarrhoea accounted for more than 25% of diarrhoeal deaths in Bangladesh, Ethiopia and Uganda. The highest proportion of diarrhoeal deaths due to acute bloody diarrhoea was observed in Bangladesh: 27.8% (5/18) in children aged 1 to 4 years. The proportion in the United Republic of Tanzania was also high: 15.4% (6/39) in infants aged 1 to 11 months and 17.1% (7/41) in children aged 1 to 4 years. At all other sites, bloody diarrhoea accounted for less than 15% of diarrhoeal deaths in infants aged 1 to 11 months and less than 10% in children aged 1 to 4 years. Acute watery diarrhoea was the commonest cause of diarrhoeal deaths at several sites: among infants aged 1 to 11 months, it accounted for 85.4% (35/41) of deaths in Bangladesh and 78.6% (114/145) in Ghana; among children aged 1 to 4 years, it accounted for 70.7% (29/41) in the United Republic of Tanzania and 69.4% (68/98) in Pakistan.

**Table 4:** Type of diarrhoea in review of childhood diarrhoeal deaths, population-based studies in seven countries, 2000–2012

Diarrhoeal deaths and type of diarrhoea	No. of children who died from diarrhoea, (%)						
	Bangladesh	Ethiopia	Ghana	India	Pakistan	Uganda	United Republic of Tanzania
**Study period, year range**	2003–2011	2003–2012	2003–2008	2008–2010	2006–2007	2007–2010	2000–2011
**Infants aged 1 to 11 months**							
Diarrhoeal deaths, no.	41	28	145^a^	197	220	28^a^	39
Persistent diarrhoea	4 (9.8)	17 (60.7)	21 (14.5)	85 (43.1)	66 (30.0)	9 (32.1)	14 (35.9)
Acute watery diarrhoea	35 (85.4)	9 (32.1)	114 (78.6)	99 (50.3)	143 (65.0)	17 (60.7)	19 (48.7)
Acute bloody diarrhoea	2 (4.9)	2 (7.1)	10 (6.9)	13 (6.6)	11 (5.0)	2 (7.1)	6 (15.4)
**Children aged 1 to 4 years**							
Diarrhoeal deaths, no.	18	32	NA	NA	98	49^a^	41
Persistent diarrhoea	5 (27.8)	18 (56.3)	NA	NA	18 (18.4)	22 (44.9)	5 (12.2)
Acute watery diarrhoea	8 (44.4)	10 (31.3)	NA	NA	68 (69.4)	20 (40.8)	29 (70.7)
Acute bloody diarrhoea	5 (27.8)	4 (12.5)	NA	NA	12 (12.2)	7 (14.3)	7 (17.1)

NA: not available.

^a^ The number does not correspond to that in Table 3 because data on the type of diarrhoea were not available for all children who died from diarrhoea.

Over 40% of children who died from persistent diarrhoea were severely malnourished in all countries except the United Republic of Tanzania: among infants aged 1 to 11 months, 90.6% (77/85) in India and 85.7% (18/21) in Ghana were malnourished; among children aged 1 to 59 months, 55.6% (5/9) in Bangladesh and 71.0% (22/31) in Uganda were malnourished (Table 5). In our analysis we found that, at the majority of surveillance sites, more than half the children who died from persistent diarrhoea had received oral rehydration solutions or intravenous fluids. The rate was slightly lower for acute diarrhoea. Rates were considerably lower in Ethiopia, where only 5.7% (2/35) of children who died from persistent diarrhoea and 15.8% (3/19) who died from acute diarrhoea had received fluids (Table 5). The proportion of caretakers who reported seeking care for their child with persistent diarrhoea ranged from 70% to 100% in all countries except Pakistan, where it was 42.9% (36/84). In addition, over 75% sought care for acute diarrhoea at five of the seven sites. There was no substantial difference in the proportion who sought care for boys or girls.

**Table 5:** Malnutrition, fluid administration and care-seeking behaviour in review of childhood diarrhoeal deaths, population-based studies in seven countries, 2000–2012

Variable	% of children						
	Bangladesh	Ethiopia	Ghana	India	Pakistan	Uganda	United Republic of Tanzania
**Age range of children, months**	1–59	1–59	1–11	1–11	1–59	1–59	1–59
**Severe malnutrition present**							
Children with persistent diarrhoea	55.6 (5/9)	42.9 (15/35)	85.7 (18/21)	90.6 (77/85)	41.7 (35/84)	71.0 (22/31)	10.5 (2/19)
Children with acute watery or bloody diarrhoea	28.0 (14/50)	24.0 (6/25)	47.6 (59/124)	47.3 (53/112)	24.4 (57/234)	52.1 (24/46)	9.8 (6/61)
**Oral rehydration solutions or intravenous fluids given**							
Children with persistent diarrhoea	NA	5.7 (2/35)	71.4 (15/21)	54.1 (46/85)	82.1 (69/84)	61.3 (19/31)	57.9 (11/19)
Children with acute watery or bloody diarrhoea	NA	16.0 (4/25)	64.5 (80/124)	38.4 (43/112)	65.0 (152/234)	50.0 (23/46)	39.3 (24/61)
**Care sought outside the home**							
Children with persistent diarrhoea	100.0 (9/9)	80.0 (28/35)	71.4 (15/21)	98.8 (84/85)	42.9 (36/84)	80.6 (25/31)	78.9 (15/19)
Children with acute watery diarrhoea	81.4 (35/43)	89.5 (17/19)	66.7 (76/114)	73.7 (73/99)	40.8 (86/211)	86.5 (32/37)	60.4 (29/48)
Children with acute bloody diarrhoea	100.0 (7/7)	100 (6/6)	60.0 (6/10)	100.0 (13/13)	43.5 (10/23)	88.9 (8/9)	61.5 (8/13)
All boys	100.0 (29/29)	84.8 (28/33)	68.2 (58/85)	93.2 (41/44)	93.3 (14/15)	77.6 (38/49)	69.2 (18/26)
All girls	92.9 (26/28)	82.1 (23/28)	68.3 (41/60)	91.4 (85/93)	91.6 (22/24)	74.1 (40/54)	66.7 (8/12)

NA: not available.

## Discussion

Our study showed that 49–85% of diarrhoeal deaths in infants aged 1 to 11 months were due to acute watery diarrhoea at six of the seven study sites, whereas 5–15% of diarrhoeal deaths at all sites were due to acute bloody diarrhoea and 10–61% at all sites were due to persistent diarrhoea (Table 4). These rates were similar to those reported in 1993, which showed that acute diarrhoea accounted for 28% of diarrhoeal deaths among infants and that persistent diarrhoea accounted for 62% in infants less than 11 months of age in Brazil.^22^ Moreover, persistent diarrhoea accounted for more than 30% of diarrhoeal deaths in infants aged 1–11 months at five of the seven sites and more than 25% of deaths in children aged 1–4 years at three of the five sites where data were available. While there is evidence that persistent childhood diarrhoea has decreased, our data shows that in some countries it is a major contributor to diarrhoeal deaths.^9^

In agreement with previous reports,^23^^–^^25^ we found that more than 50% of children who died from persistent diarrhoea were malnourished at four of the seven study sites; the proportion was over 70% at three sites. However, the relationship between diarrhoea, particularly persistent diarrhoea, and malnutrition is bidirectional and it is not possible to determine the extent to which malnutrition may be due to persistent diarrhoea.^26^^,^^27^

Surprisingly, at six of the seven sites, 70–100% of children with persistent diarrhoea who died had been seen in a health-care facility. Although the cause of death was probably multifactorial, this finding raises questions about the quality of the care provided. Mortality could be reduced by improving the quality of care and by increasing awareness of the need for immediate treatment. Further research is needed on how best to manage persistent diarrhoea in low- and middle-income countries. Furthermore, at most sites we found that more than half of children with persistent diarrhoea who died had received oral rehydration solutions or intravenous fluids, though we had no data on the volume of fluids administered or the duration of treatment. The rate of fluid use was lower for acute diarrhoea than for persistent diarrhoea, perhaps because of the shorter disease duration. Previous studies have shown that the correct use of oral rehydration solutions is uncommon in cases of acute diarrhoea.^28^^,^^29^ In addition, data from demographic and health surveys have shown that the use of oral rehydration solutions or zinc is low in many countries, though there is a great variation.^12^^,^^13^^,^^15^^–^^19^Although the use of oral rehydration, zinc and antibiotics for bloody diarrhoea needs to be scaled up,^30^ doing so might not be sufficient to reduce diarrhoeal deaths and achieve the target set by the Global Action Plan for the Prevention and Control of Pneumonia and Diarrhoea,^10^ which is that mortality from diarrhoea in children aged less than 5 years of age should be less than 1 per 1000 live births.

### Limitations

We used data on diarrhoeal mortality collected at the seven sites by verbal autopsy, in which the cause of a child’s death, or the sequence of causes that led to death, is determined from information obtained by interviewing the next of kin or other caregivers using a standardized questionnaire.^14^ Although this approach is frequently used in public health research for collecting mortality data at a community or population level, it may not be an accurate way of attributing the cause of death in individuals. However, since our data came from population-based cohorts, they provide more information on the pattern of deaths in the general population than hospital data. Nevertheless, the small size of the study population at some sites may have influenced interpretation of the findings at those sites. In addition, even though we restricted the analysis to the time period between 2000 and 2012, there was still some variability in the age of the data among sites, which may have affected our interpretation of the clinical patterns observed. We were not able to investigate the effect of potentially important variables such as HIV infection as most sites did not collect the information needed. Finally, given the variability between sites in the data collected, we were not able to compare sites.

Our findings indicate that a greater focus on the treatment of persistent diarrhoea is needed. In particular, if most children with persistent diarrhoea are moderately or severely malnourished, treatment might have to include therapeutic foods. Nearly two decades ago, an International Working Group on Persistent Diarrhoea developed a treatment algorithm based on the findings of a multicentre cohort study.^31^ Although use of the algorithm was recommended internationally, a recent consultation on diarrhoea by WHO concluded that it had been implemented in very few places and that there was a need for a policy to promote its wider use in treatment and research.^32^

In conclusion, we found that persistent diarrhoea accounted for a substantial proportion of diarrhoeal deaths in young children in low- and middle-income countries. However, many global and national strategies and initiatives for the prevention and treatment of diarrhoea in children do not mention persistent diarrhoea. Moreover, little research on the condition has been carried out over the last two decades. If the Global Action Plan for the Prevention and Control of Pneumonia and Diarrhoea target for reducing mortality from diarrhoea is to be achieved,^10^ public health policies must be changed to include the management of persistent diarrhoea. In addition, research is needed into the public health burden of persistent diarrhoea and barriers to the implementation of recommended treatment to understand why children are still dying from the condition.
